# The prevalence of gastrointestinal symptoms among patients with COVID‐19 and the effect on the severity of the disease

**DOI:** 10.1002/jgh3.12415

**Published:** 2020-09-18

**Authors:** Tharwat Sulaiman, Ali A Algharawi, Marwan Idrees, Rafid H Alzaidy, Kawthar Faris, Graham Cullingford, Jawad Rasheed

**Affiliations:** ^1^ Department of Surgery College of Medicine, University of Baghdad Baghdad Iraq; ^2^ Department of Surgery Baghdad Teaching Hospital Baghdad Iraq; ^3^ General Surgery Fiona Stanley Hospital Perth Western Australia Australia; ^4^ Medical administration Baghdad Teaching Hospital Baghdad Iraq; ^5^ TDM Centre Baghdad Teaching Hospital Baghdad Iraq; ^6^ Department of Surgery University of Western Australia Perth Western Australia Australia

**Keywords:** COVID‐19, gastrointestinal symptoms, mortality, severity

## Abstract

**Background and Aim:**

COVID‐19 is a new pandemic disease recognized by the World Health Organization. It mainly affects the respiratory system, but it can also affect other systems. The gastrointestinal system has been found to be affected in many patients. This study investigated the COVID‐19‐related gastrointestinal manifestations and the effect of gastrointestinal involvement on the course and outcome of the disease.

**Methods:**

This was a retrospective descriptive study conducted on 140 COVID‐19 polymerase chain reaction‐positive symptomatic individuals admitted to Al‐Shafa Hospital – Medical City Complex in Baghdad, Iraq during the period 2 March 2020 to 12 May 2020. Demographic data and clinical presentation and laboratory data were extracted from the case sheets of the patients and were also obtained from direct communication with the patients, their families, and medical staff.

**Results:**

Gastrointestinal (GI) symptoms alone were detected in 23.6% of the patients; 44.3% of the patients presented with only respiratory symptoms, and 32.1% presented with both respiratory and GI symptoms. Patients with only GI symptoms had less severe disease compared with those who had both GI and respiratory symptoms, who had more severe disease with higher mortality. Overall mortality was 8.6%, with no mortality in the GI symptoms alone group. The highest severity and mortality were in patients with both GI and respiratory symptoms (48.39 and 13.33%, respectively).

**Conclusions:**

COVID‐19‐related gastrointestinal symptoms are common, and their presence alone carries a better prognosis, but their presence with respiratory symptoms is associated with higher morbidity and mortality.

## Graphical abstract

This study investigated the COVID‐19‐related gastrointestinal manifestations and the effect of gastrointestinal involvement on the course and outcome of the disease. We concluded that COVID‐19‐related gastrointestinal symptoms are common, and their presence alone carries a better prognosis, but their presence with respiratory symptoms is associated with higher morbidity and mortality.

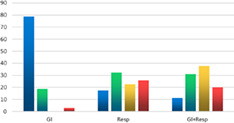

## Introduction

A novel corona virus appeared in Wuhan, China, in December 2019, subsequently named Severe Acute Respiratory Syndrome Corona Virus (SARS‐CoV‐2).^1^ It was responsible for an outbreak of pneumonia called corona virus disease 2019 (COVID‐19), which started in China, becoming a pandemic and paralyzing society all over the world.^1^, ^2^, ^3^, ^4^, ^5^

COVID‐19 most commonly presented with respiratory symptoms, such as cough and shortness of breath.^6^ However, it can present with nonrespiratory symptoms such as gastrointestinal (GI) symptoms in the form of diarrhea, loss of appetite, and nausea.^7^, ^8^, ^9^, ^10^, ^11^, ^12^, ^13^

GI symptoms are common, and for many patients, their initial symptoms may be diarrhea and/or vomiting rather than respiratory symptoms.^9^, ^12^ Nearly half of patients with COVID‐19 might present with GI symptoms in addition to fever and/or respiratory symptoms. Specifically, DIARRHEA frequency varies from 3 to 40% of the patients.^11^ GI symptoms may be present even before onset of fever or respiratory symptoms.^12^, ^13^ An important observation regarding the duration of the disease is that patients with digestive symptoms have a longer duration from onset to admission to the emergency department, which may make them more infective during this period.^12^, ^13^ Digestive symptoms are common in the community, mostly not due to COVID‐19, but still, one should be alert and consider the possibility of COVID‐19 as a cause.^12^, ^13^

This study is intended to identify the incidence of digestive symptoms in a sample of Iraqi patients in a single center who have COVID‐19 and discuss the behavior of the disease in those patients.

## Methods

This is a descriptive retrospective analysis of data collected from a single center in Baghdad, Iraq, from patients (positive, symptomatic patients) diagnosed as confirmed cases of COVID‐19 based on a positive polymerase chain reaction (PCR) test performed as investigation due to their symptoms or their exposure to a previously known COVID‐19‐positive patient.

These proven PCR‐positive individuals were admitted for isolation and treatment, if subsequently needed, to the Medical City Complex (Al Shafa Hospital) between 2 March 2020 and 12 May 2020. These individuals were then closely interrogated for symptoms, and symptomatic patients were analyzed for this study.

The demographic data, symptoms and signs at presentation, and other clinical and laboratory data were evaluated with special consideration of GI manifestations in those patients. These data were collected from the case sheets of the patients, from direct communication with the patients or their relatives, and from attending doctors and medical staff in order to complete the data missed in the case sheets. All data were input into an Excel spreadsheet. Patients were classified according to the involved system into three groups: those who presented with only GI symptoms (GI group), those with respiratory symptoms alone (Resp. group), and those with both respiratory and GI symptoms (Resp and GI group).

The severity of the disease was classified as:

1. Mild cases (no pneumonia on computed tomography [CT] scan)
2. Moderate cases (pneumonia on CT scan)
3. Severe cases (pneumonia on CT scan) with multiorgan failure.
4. Critical cases requiring admission to the intensive care unit (ICU).

The GI group was classified as mild or severe depending on whether there was a subsequent admission to ICU.

Statistical analysis was done using SPSS.23 including chi‐square and F‐test by anova, and a *P*‐value = or < 0.05 was considered significant.

Approval was obtained from the ethical committee of the Medical City Complex and the Scientific Committee of Corona Virus Disease in the Iraqi Ministry of Health.

## Results

Of individuals diagnosed as positive by PCR following screening due to exposure to known patients with COVID‐19 or presence of suspicious symptoms, 170 people had symptoms. Of these, 30 patients were excluded from analysis due to incomplete data, resulting in 140 patients being included in the final analysis.

A total of 98 (70%) were patients from the local Baghdad population with no history of travel, 31 (22%) had returned from a visit to Iran, and 11 (8%) were medical workers with known exposure to COVID‐19 patients. No population surveillance was being performed at that time.

The average age at presentation was 45 ± 17 years, with no statistical difference between those with only respiratory symptoms (Resp group), those with only GI symptoms (GI group), and those who had both respiratory symptoms and GI symptoms (Resp and GI group) (Table 1). The male‐to‐female ratio was 2.5:1, with a greater male preponderance in the GI group (3.7:1, *P* = 0.048).

**Table 1:** Baseline patient characteristic based on the classification of presenting symptoms in COVID‐19 patients

Item	Total	Digestive only	Respiratory only	Digestive + respiratory	*P* value	Chi‐square
Age (years)	44.99 ± 16.81	41.09 ± 19.36	45.87 ± 15.5	46.64 ± 16.49	0.3065	
					NS	
Weight\kg	82.12 ± 8.54	79.84 ± 14.3	83.03 ± 5.51	82.55 ± 5.82	0.2052	
					NS	
Gender, *n* (%)	F = 40 (28.57%)	F = 7 (21.2%)	F = 17 (27.41%)	F = 16 (35.5%)	<0.0001	20.28
	M = 100 (71.42%)	M = 26 (78.7%)	M = 45 (72.58%)	M = 29 (64.4%)		
Fever	112 (80%)	22 (66.66%)	46 (74.19%)	44 (97.78%)	0.0010	13.86
Temperature	38.23 ± 0.64	38.08 ± 0.63	38.28 ± 0.69	38.34 ± 0.57	0.1904	
Digestive symptoms						
Abd. pain	42 (30%)	20 (60.6%)	0 (0.0%)	22 (48.80%)	<0.0001	48.94
Diarrhea	41 (29.28%)	16 (48.4%)	0 (0.0%)	25 (57.70%)	<0.0001	46.55
Vomiting	31 (22.14%)	14 (42.4%)	0 (0.0%)	17 (37.77%)	<0.0001	31.89
Loss of appetite	40 (28.57%)	21 (63.6%)	0 (0.0%)	19 (44.40%)	<0.0001	32.03
Respiratory symptom						
Cough	90 (64.28%)	0 (0.0%)	52 (83.87%)	38.(84.40%)	<0.0001	77.72
Exertional dyspnea	25 (17.85%)	0 (0.0%)	17 (41%)	8 (17.70%)	0.0040	11.04
SOB	74 (52.85%)	0 (0.0%)	39.(62.90%)	35 (77.70%)	<0.0001	50.73
Sore throat	49 (35%)	0 (0.0%)	27 (43.58%)	22 (48.80%)	<0.0001	23.58
Sneezing	10 (7.14%)	0 (0.0%)	5 (8.06%)	5 (11.10%)	0.1583	3.686
					NS	
Runny nose	6 (4.28%)	0 (0.0%)	4 (6.45%)	2 (2.20%)	0.3346	2.189
					NS	
Loss of smell or taste	2 (1.42%)	0 (0.0%)	2 (3.22%)	0 (0.0%)	0.5093	1.350
					NS	
Constitutional symptoms						
Headache	75 (53.57%)	13 (39.30%)	31 (50%)	31 (68.80%)	0.0269	7.230
Chest tightness	25 (17.85%)	3 (9.09%)	9 (14.51%)	13 (28.80%)	0.0515	5.934
					NS	
Tiredness	48 (34.28%)	7 (21.2%)	16 (25.81%)	25 (55.50%)	0.0012	13.52
Agitation	6 (4.28%)	2 (6.06%)	3 (4.83%)	1 (2.20%)	0.6816	0.7668
					NS	
Presenting comorbidity						
HT	34 (24.28%)	0 (0.0%)	15 (24.19%)	19 (42.22%)	<0.0001	18.46
DM	32 (22.85%)	3 (9.09%)	16 (25.80%)	13 (33.33%)	0.0916	4.781
					NS	
SHD	8 (5.33%)	0 (0.0%)	1 (1.6%)	7 (15.50%)	0.0024	12.02
SLE	1 (0.71%)	0 (0.0%)	0 (0.0%)	1 (2.20%)	0.3454	2.126
					NS	
Asthma	8 (5.71%)	0 (0.0%)	5 (8.06%)	3 (6.66%)	0.2578	2.711
					NS	
Organ transplant	1 (0.71%)	0 (0.0%)	1 (1.6%)	0 (0.0%)	0.5307	1.267
					NS	
Process (days)						
Starting signs and symptoms	5.24 ± 2.58	4.60 ± 1.80	5.19 ± 2.61	5.77 ± 2.94	0.1388	
					NS	
Hospital stay	13.07 ± 5.36	12.36 ± 3.73	13.17 ± 4.97	13.46 ± 6.77	0.6605	
					NS	
CT scan positive	88 (62.85%)	3 (9.09%)	47 (75.89%)	38 (84.44%)	<0.0001	54.30
X‐ray positive	85 (60.71%)	4 (12.1%)	47 (75.89%)	34 (75.55%)	<0.0001	42.75

Statistical method: Use SPSS.23 for statistical analysis, to result chi‐square and F‐test by anova by using *P*‐value. If *P* < 0.05, significant. If *P* > 0.05, nonsignificant. If *P* < 0.001, high significant.

CT, computed tomography; DM, diabetes melletus; GI, gastrointestinal; HT, hypertension; SHD, structural heart disease; SOB, shortness of breath.

**Figure 1:**
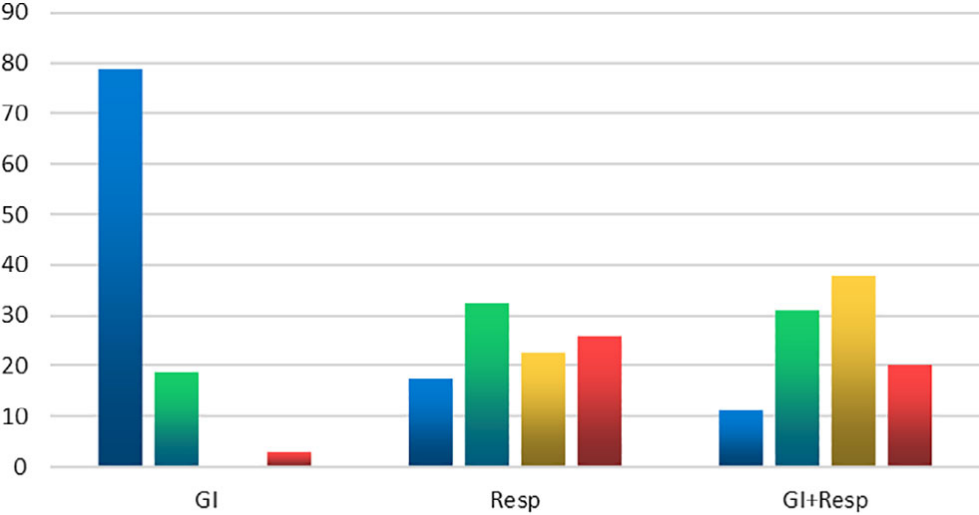
Classification of the patients according to severity of illness. GI, gastrointestinal. (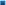), Mild; (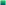), moderate; (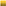), severe; (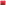), critical.

Thirty‐three patients (24% of the 140 patients) presented with one or more GI symptoms only, 62 patients (44%) presented with only respiratory symptoms, and 45 patients (32%) presented with both respiratory and digestive symptoms (please refer to Fig. 1).

Fever, defined as a temperature of >38 °C, was recorded in 55% of patients (77); fever was greater among patients who had both respiratory and GI symptoms.

GI symptoms were variable; abdominal pain was recorded in 42 patients (30%), diarrhea in 41 patients (29%), loss of appetite in 40 patients (29%), and vomiting in 32 patients (22%) (please refer to Table 1).

Most of the patients with only GI symptoms had no comorbidity.

The mean duration of hospital stay was 13 days with no statistically significant difference between all three groups (*P* = 0.666) (Table 1).

Although those patients within the GI group are expected to have negative CT chest or chest X‐ray findings, but there were 3 (9%) who had positive CT chest findings despite no respiratory symptoms.

The majority of patients presented with mild (42 patients 30%) or moderate (41 patients 29%) symptoms and signs; severe and critical conditions constituted around 41% (Fig. 1). Those with only GI symptoms usually presented with mild (26 patients 79%) or moderate (6 patients 19%) symptoms. Only one patient from the GI group presented with a severe or critical condition (*P* = 0.0001), while 26 (18.5%) of the Resp and GI group had severe or critical condition (*P* = 0.0059) (Fig. 1).

Patients in the Resp group and the GI and Resp group were more likely to have severe or critical illness (48% of the Resp group and 58% of those with both Resp and GI symptoms). (Fig. 1).

**Figure 2:**
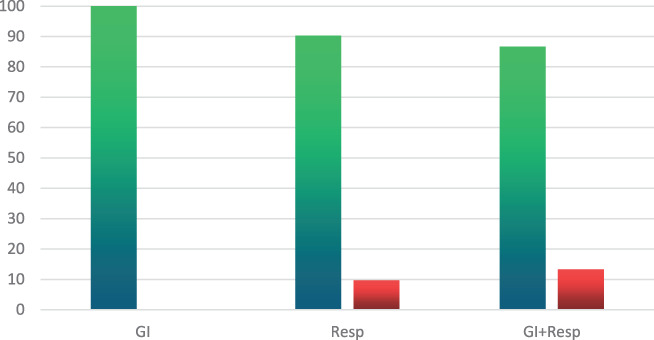
The final outcome (complete recovery or death) of all 140 patients. (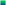), Complete recovery; (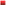), death.

All the GI group patients recovered with no residual effects, compared with a 90% recovery rate in the Resp. group and 87% among those in the respiratory and GI group (Chi‐square = 4.49, *P* = 0.105) (please refer to Fig. 2).

Total mortality among the 140 patients was 8.6% (12 patients), with no recorded mortality in the GI group (*P* = 0.0688). Six patients (10%) died in the respiratory group, and another six patients (14%) died in the respiratory plus GI group (Fig. 2).

**Figure 3:**
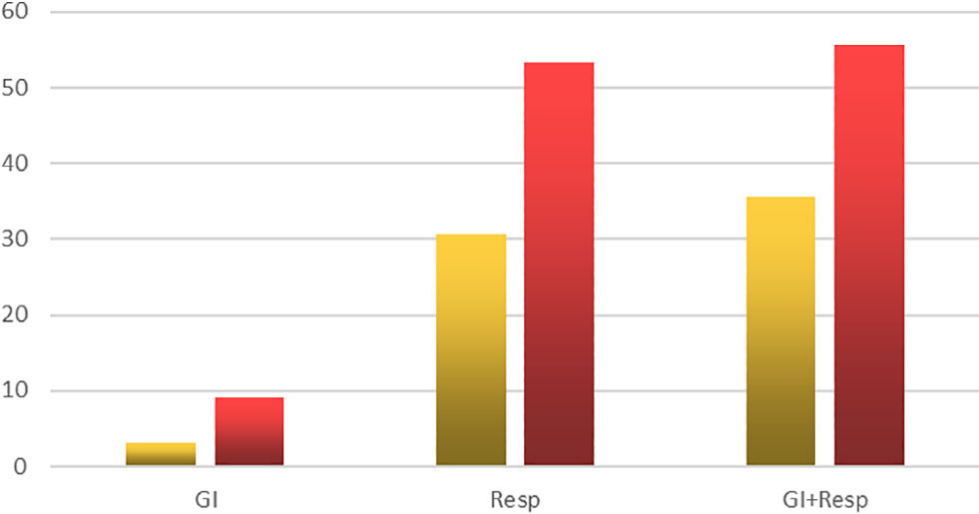
Requirement for Respiratory care unit admission or the need for oxygen therapy. GI, gastrointestinal. (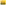), RCU (Respiratory Care Unit); (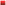), oxygen supply.

We noticed that those patients who presented with GI symptoms only usually had mild or moderate disease, and only one patient required admission to the ICU (*P* = 0.0002). Those in the Resp group had more severe disease requiring admission to the ICU in 30.6%, while those with both Resp and GI symptoms usually presented with more severe disease, and 35.5% of them were admitted to the ICU (please refer to Fig. 3). Those with GI symptoms only had a complete recovery within the period of study, compared to 90.3% recovery in the respiratory group and 86.7% in the Resp and GI group.

## Discussion

This study was conducted in one of the first centers in Iraq to deal with COVID‐19, which was established on 1 February 2020 in Medical City Hospital. The patients were tested, and if proved to be COVID‐19 positive by PCR, they were admitted to a special hospital (Al‐Shafa) designed and equipped especially for isolation of asymptomatic PCR‐positive patients and treatment of symptomatic patients.

The aim of this study was to shed further light on the prevalence of GI symptoms, which might obscure the diagnosis of COVID‐19, thus delaying the diagnosis or leading to misdiagnosis as a medical or a surgical emergency.

COVID‐19 patients primarily present with respiratory symptoms; however, other systemic symptoms can occur as well. The etiology of GI symptoms associated with COVID‐19 is not fully understood, but ACE2 plays a critical role in the pathogenesis of the disease.^14^ The intestinal wall is invaded by COVID‐19, leading to increased permeability and easier invasion by pathogens.^15^

In an early study in one of the hospitals in Wuhan, fever was the most common presenting symptoms, followed by fatigue and dry cough, while diarrhea was recorded in only 10.1% with nausea, vomiting, and abdominal pain in much lower frequencies.^16^ At the beginning, the WHO in European regions also reported a low prevalence of GI symptoms.^17^ A later study from Wuhan reported a higher frequency of GI symptoms, increasing up to 39.6%, with nausea being the most common symptom.^18^ This report was followed by a report by Pan *et al*. from China who documented that more than 50% of their patients had GI symptoms.^9^ In a study from California, 31.9% of patients presented with GI symptoms, and the majority of these patients had mild symptoms.^19^ Another study from Zhuhai in China reported GI symptoms in only 11.6%, with diarrhea the most common symptom.^18^ For that reason, we recommend considering COVID‐19 testing in patients with new‐onset diarrhea, even in the absence of respiratory symptoms, in high‐prevalence areas.

Isolated GI symptoms in COVID‐19 patients have been reported.^7^, ^8^, ^9^ Pan *et al*. reported that 48.5% of patients in their series presented to the hospital with digestive symptoms as their main complaint.^9^ A newer study from California stated that none of their patients developed isolated GI symptoms, although 31.9% of the patients experienced concurrent GI symptoms.^19^

Contrary to other studies,^9^, ^13^, ^15^ where patients with digestive symptoms had a significantly longer time from onset to hospital attendance, we found that patients with only GI symptoms presented earlier than those with respiratory symptoms, which could be related to our health system, where patients can attend any hospital or medical center for treatment, even for mild diseases.

As the disease was mainly mild or moderate, we did not observe any mortality among patients with only GI symptoms. Mortality was the highest among patients with both respiratory and GI symptoms.

The average age of patients in this study was 44 years. There was no difference in relation to the presence or absence of GI symptoms. In this study, the patients were much younger than those presented by Han *et al*. from Wuhan,^15^ where their average age was 62.5 years.

Although many previous studies reported equal gender distribution in patients who presented with GI symptoms,^13^, ^15^ we found that males were more affected than females, with a ratio of 2.5:1, with more male preponderance among those with GI symptoms alone (3.7:1). This could simply reflect a cultural issue rather than being a true epidemiological difference.

This study is important in highlighting the GI system involvement in COVID‐19 infection, which can mimic a variety of GI differential diagnoses. Not considering COVID‐19 in those patients can lead to a diagnostic dilemma and prolongation of spread of disease through delayed isolation.^15^ Delay in isolation will risk exposing hospital staff, other non COVID‐19 patients, and the patient's family members to the COVID‐19 virus. Furthermore, not recognizing COVID‐19 infection in patients with only GI symptoms can lead to underestimation of virus incidence and therefore lead to less adequate transmission prevention.

In some cases, GI symptoms appeared before fever or respiratory symptoms. We found in our study that the GI symptoms appeared at around 4.6 days from exposure to a COVID‐19 patient compared with the average of 5.2 days for the respiratory symptoms to appear.

In our center, we perform PCR testing for COVID‐19 for any patient planned for elective or semiurgent surgery. For patients requiring emergency surgery, all undergo COVID‐19 PCR testing; if PCR positive, patients will be isolated with COVID‐19 precautions, and the whole involved staff will be isolated until their COVID‐19 PCR results are known. Furthermore, three proven COVID‐19‐positive patients in any ward led to ward closure for appropriate decontamination protocols.

The presence of digestive symptoms might indicate a higher viral load, which adds to the severity of illness.^9^, ^15^, ^19^ The presence of the virus in the stool should raise the possibility of fecal–oral transmission, and protective measures should be taken.^13^, ^20^

This study has its limitations, which includes its retrospective design that can lead to recall errors; furthermore, the relatively small population number may not be a true reflective of the general population. Further larger population epidemiologic studies are recommended for further confirmation of our findings.

In conclusion, GI symptoms are common in people with COVID‐19 infection. COVID‐19 patients with pure GI symptoms are more likely to have a more indolent and mild disease, which can delay their presentation to hospital and, subsequently, their diagnosis. Investigating for COVID‐19 in the presence of concerning GI symptoms should be considered for earlier diagnosis and potentially better prognosis.
